# Study on Early Onset Melanoma and Germ-Line Mutation in CDKN2A among Patients in Imam Khomeini Hospital Complex

**DOI:** 10.31557/APJCP.2021.22.10.3347

**Published:** 2021-10

**Authors:** Samira Ferdosi, Mojtaba Saffari, Razieh Alishahi, Alireza Ghanadan, Reza Shirkoohi

**Affiliations:** 1 *Cancer Research Center, Cancer Research Institute, Imam Khomeini Hospital Complex, Tehran University of Medical Sciences (TUMS), Tehran, Iran. *; 2 *Department of Medical Genetics, School of Medicine, Tehran University of Medical sciences Cancer Research Center, Cancer Institute of Iran, Tehran University of Medical Sciences, Tehran, Iran. *; 3 *Department of Dermatopathology, Razi Hospital, Tehran, Iran. *

**Keywords:** Melanoma, CDKN2A, familial cases, mutation

## Abstract

**Objective:**

Malignant melanoma is a highly lethal melanocytic neoplasia with different predisposing factors. The genetic background in familial cases is an important issue in finding at risk family members. CDKN2A is one of these predisposing genes which have been estimated to be involved in germ line mutation in approximately 5-10% of familial melanoma cases.

**Materials and Methods:**

An inclusion criteria for familial melanoma was prepared according to the literature, and the age of onset was considered as a single criteria for selection. A total number of 322 melanoma cases were investigated regarding the criteria, among which 20 patients were chosen (<40 years). DNA was extracted from Formalin Fixed Paraffin Embed of normal tissues and DNA sequencing was performed for all coding sequences of CDKN2A (p16).

**Results:**

One of the cases showed a pathogenic mutation in codon 108, exon 2(322G >C; Asp108His). Further analysis of his offspring indicated no mutation in the next generation.

**Conclusion:**

**A**s far as the authors of the present study are concerned, this was the first report on this germ-line mutation with mentioned amino acid alteration in the melanoma. Screening the CDKN2A gene for possible mutation could prevent the incidence of familial cases in at risk members.

## Introduction

**Figure 1:**
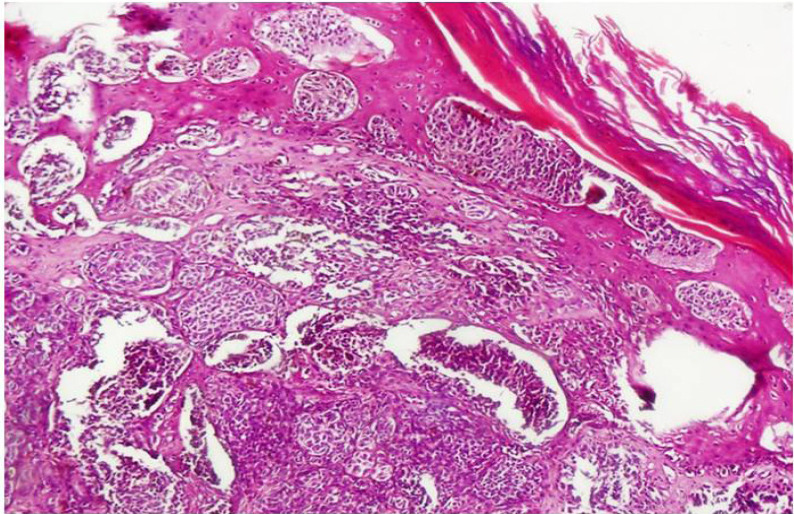
Malignant Melanoma with Pagetoid Distribution of Melanoma Cells and Consumption of Epidermis in Right Side of the Field (H&E, magnification × 20)

Cutaneous malignant melanoma (CMM) is a malignant skin tumor; it is a heterogeneous ailment with different environmental and genetic factors (Hill et al., 2013; Yang et al., 2015). Melanoma is a malignant tumor of melanocytes (Ferdosi et al., 2016), (Figure 1) which is often considered one of the most lethal and treatment resistant human cancers (Louveau et al., 2020). Its incidence rate is enhancing significantly (Rhee et al, 2013) and it is the reason for vast majority of skin cancer deaths all over the world (Thompson et al., 2009). The number of new cases and deaths of melanoma in US are reported to be 76,380 and 10,130, respectively (Siegel et al., 2016). The highest incidence rate of melanoma is in Australia (38.5/100,000 for men and 29.5/100,000 for women), the medium rate is in North America (16.4/100,000 for men and 11.7/100 000 for women), and the lowest rate is in Western Europe (7.3/100,000 for men and 10/100,000 for women). 

A positive family history associates with intensifying risk of melanoma; approximately 10% of melanoma occurs in a familial setting with CDKN2A mutations (Rossi et al, 2019; Read et al, 2016). The two genes with high risk susceptibility for CMM are called CDKN2A and CDK4 (Puntervoll et al., 2013). These genes are found to be mutated in up to 40% of familial melanomas and approximately 2% of all melanoma cases (Lee et al., 2015). Mutations in CDKN2A has the lowest frequency in Australia (20%) and the highest frequency in Europe(57%) (Karagianni et al., 2018). Cyclin-dependent kinase inhibitor 2A (CDKN2A), the first known melanoma susceptibility gene, is located on chromosome 9 (9p21.3) (Ming et al., 2020). 

CDKN2A encodes two separate proteins, p16INK4A (p16) and p14ARF (p14), both of which are involved in cell cycle inhibition via various pathways (Chan et al., 2021). CDKN2A is known as the redundant high penetrance human familial melanoma locus. The incidence rate of germ-line mutation in CDKN2A is very low in general population (Aoude et al., 2015). Germ-line CDKN2A mutations are found in 5-20% of familial melanoma cases (Helgadottir et al., 2014, Helgadottir et al., 2015). Furthermore, this mutation is associated with enhanced risk of melanoma (Helgadottir et al., 2016). It has been seen that germ-line mutations in p16 have currently caused a wide range of cancers like melanoma and pancreatic carcinoma; it has also been reported that there is a remarkable association between this mutation(CDKN2A) and pancreatic neoplasia (Jenkins et al., 2013; Potrony et al., 2015). The researchers of the present study have shown in their previous study that the majority of patients are diagnosed in higher stages of disease (Ferdosi et al., 2016); thereafter, finding methods to reduce the rate of advanced melanoma is of great importance. Screening familial cases which are eligible to do the mutation analysis is one of the critical policies in this regard. Therefore, the present study involved cases which followed CDKN2A mutation analysis. 

## Materials and Methods

*Patients and tissues*

Cases were selected based on patient’s file review from archives of the Cancer Institute of Iran, Imam Khomeini Hospital Complex, Tehran University of Medical Sciences, Tehran, Iran. An inclusion criteria was prepared based on literature and patients were selected to participate in the study based on the age of onset (Table 1) (Razi et al., 2015; Sa et al., 2018). Formalin fixed paraffin embedded (FFPE) blocks obtained from selected patients were observed by pathologist and normal tissue was separated for DNA extraction and germline mutation analysis. Out of a total number of 322 patients, 20 met the inclusion criteria and entered the study for FFPE block selection and DNA extraction. 

*Preparation of DNA*

DNA extraction was performed by QIAamp DNA mini kit (Qiagen, USA) according to recommended protocol. First, paraffin was removed according to manufacturer’s instruction, and then DNA was extracted from tissue samples using mentioned DNA extraction kit. The quantity and quality of extracted DNA were measured using NanoDrop (ND2000, thermoscience, USA) and agarose gel electrophoresis, respectively 

*PCR, Sequencing, and analysis*

PCR was implemented on DNA extractions and specific primers were employed for all exons (exons1, 2, and 3) of CDKN2A (Table-2) using PCR master mix (1.5mM MgCl2) (Ampliqon, Denmark). The Standard PCR amplification conditions consisted of a hot start at 94°C for 5 minutes, followed by 94°C for 30 seconds, 62°C for 30 seconds, and 72°C for 30 seconds, for 30 to 40 cycles, with a final amplification at 72°C for 5 minutes. DNA quality was confirmed using DNA staining by GelRED (Biotum- US) on a 2% agarose gel and a band of 500bp was observed. For mutation detection, the researchers analyzed PCR products using Sanger DNA sequencing (ABI 3130, USA). Both forward and reverse PCR sequencing data from each sample were monitored using FinchTV 1.4.0 software (Geospiza, USA) for any altered nucleotide arrangement and were aligned with the normal sequence of CDKN2A using BLAST, from NCBI bioinformatics tool (https://blast.ncbi.nlm.nih.gov/Blast.cgi). The possible alteration was checked for probability of mutation or polymorphism using Mutation Taster and Clinvar online tools. The protein sequence was analyzed for domain information and the position of mutation was identified based on the function of protein motifs using the Eukaryotic Linear Motif (ELM) resource for Functional Sites in Proteins (http://elm.eu.org/). 

A complete three generation pedigree was drawn for a patient with mutation and his family members were invited for screening of the same germ-line mutation (consent forms were obtained from all participants). DNA was derived from fresh blood and DNA sequencing was done for the same mutation.

## Results

*Sample preparation*

A total number of 322 cases (163 males and 159 females) selected from archives of Cancer Institute of Iran were investigated in the present study. The patients were within the age range of 25-75 with the average age of 55. Out of 322 patients, 20 met the inclusion criteria (i.e., the age of onset).

*PCR sequencing analysis*

**Figure 2:**
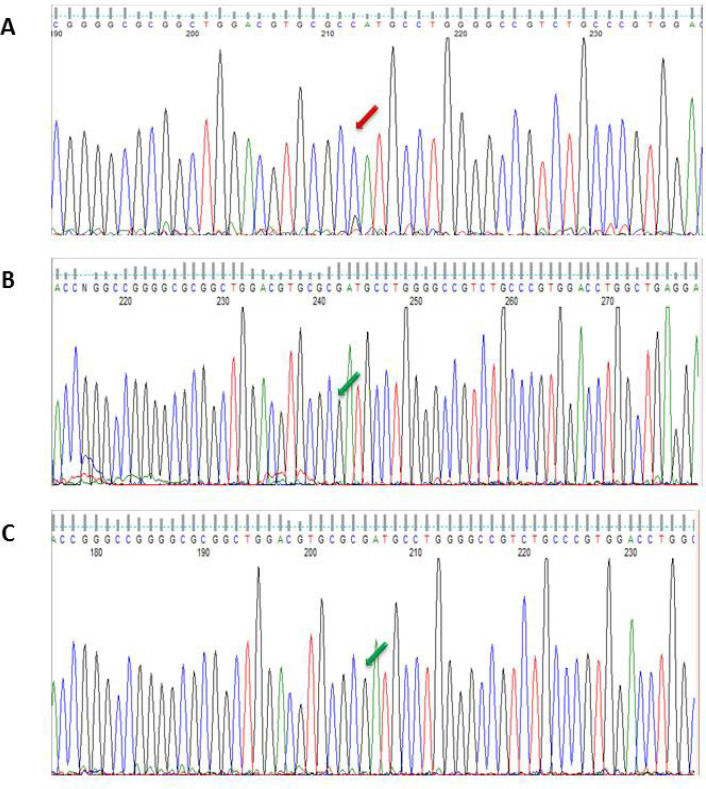
DNA Sequence Analysis. The c.322G>C; Asp108His mutation detected in patient (A) which was not detected in his daughters (B, C)

**Figure 3:**
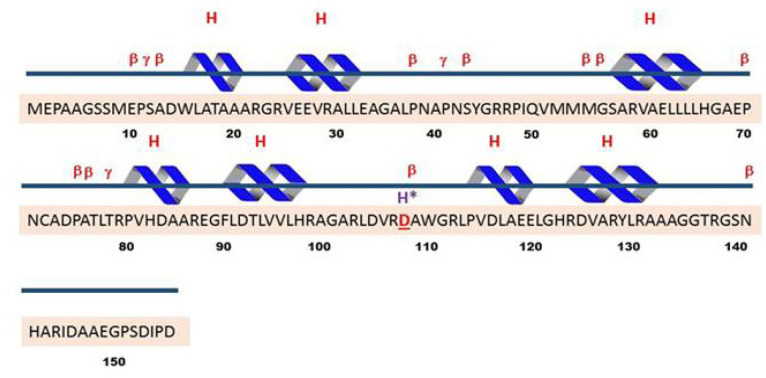
The Protein Sequence of CDKN2A. A spartate (D) located on 10th beta turns [37] which is replaced by Histidine (H*) Helices labelled H, Motifs: beta turn β, gamma turn γ

**Figure 4:**
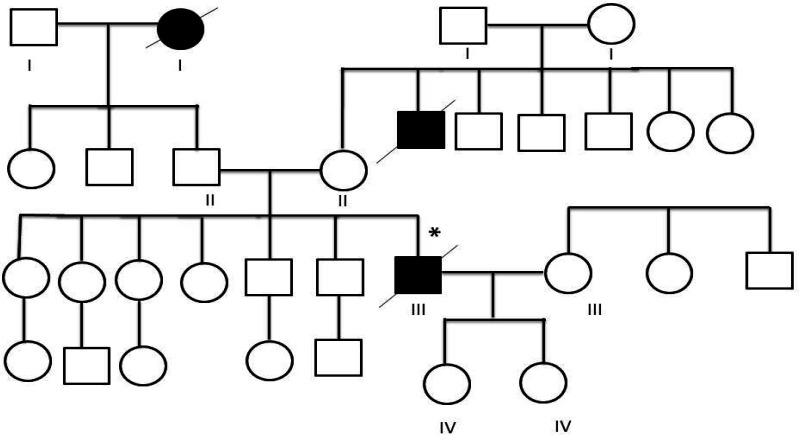
Pedigrees of Melanoma Patient with the Mutation Asp108His. III-7: Indicates tested individual, positive for the CDKN2A mutation. There was a history of gastric cancer in the family (II-5)

DNA sequencing data was analyzed to check mismatches and any alterations. There was a nucleotide change in G position as a missense change in one of cases with malignant melanoma (Figure 1) which caused amino acid alteration. The data analysis indicated mutation in exon 2 of CDKN2A gene (322G >C; Asp108His) (Figure 2 A). The missense mutation caused alteration of Aspartate amino acid to Histidine in codon number 108. Analysis of mutation data using bio-informatic tool (Mutation taster) showed pathogenic characteristic. The mentioned germ line mutation at this nucleotide side and with different amino acid alteration was found to be reported before as Uncertain and likely pathologic and in it is presented as a conflicting amongst these two mentioned category (rs121913381) for this disease in ClinVar, considering the previous literature. Searching motif activity of CDK2NA protein in the mutation point revealed a caspase activity in this position using the Eukaryotic Linear Motif (ELM) resource for Functional Sites in Proteins (Figure 3). A pedigree based on three generation was drawn for his family. Patient had a history of long time exposure to sun light (8 hours per day) in the central area of Iran which has a dry and sunny climate for 5 years due to his job responsibility as a building constructor. He had white skin, brown eyes with dark hair and belonged to one of main Iranian races (Lor). Patient had a single atypical nevus with 9 mm diameter in chest, 15 mm lateral to left nipple with vertical growth and 1.6 mm thickness. The patient had left axillary lymph node involvement and deceased. Family pedigree indicated a family history of gastric cancer in a member of his second degree relative on his mother side (Figure 4). There was no other history of cancer in all three generations. The proband had 2 daughters who were investigated for the same mutation in exon 2 of CDKN2A gene after informed consent assignment. No mutation was observed in the same position in both of them (Figure 2B, C).

**Table 1:** Including Criteria

Low melanoma incidence area ^a^	* Two primary melanoma in one person^c^ * Families with at least one of members with invasive melanoma and one or more other first- or second-degree family members with melanoma and/or pan*creatic cancer (Which should be in the same side of the family) Early onset (<40 years)
Moderate to high melanoma incidence area^b^	* Three primary melanoma in one person^c^ * Families with at least one of members with invasive melanoma and two or more other first- or second-degree family members with invasive melanoma and/or pancreatic cancer (Which should be in the same side of the family) * Early onset (<40 years)

^a^, Sistan and Baluchestan provinces in males and Hormozgan province in females; ^b^, Semnan, Isfahan, and Hamedan provinces in males and Semnan, Yazd, and Isfahan provinces in females; ^c^, could occur same time or in different period of time

**Table 2:** Information of Primers

Exon Name	Forward Sequence	Reverse Sequence
Exon 1	GGAAGAAAGAGGAGGGGC	CTGCAAACTTCGTCCTCC
Exon 2	GGGGCTCTACACAAGCTTCC	AGGGCGATAGGGAGACTCAG
Exon 3	CGTGAAGCCATTGCGAGAAC	CCCCCTGAGCTTCCCTAGTT

**Table 3:** CDKN2A Mutation Study in Familial Melanoma

Country	Group	Sample size	Number of cases with mutation	Novel mutation
Austria	Burgstaller-Muehlbacher, et al	700	4	p.A34V c.151-4 G>C
Italy	Cossu A, Casula M, et al	532	24/316(7.6%)	Gly23Asp
Queensland, Australia	Aitken J, et al	482	9/87 (10.3%)	Nt500G
Nine geographic regions in Australia, Canada, the United States, and Italy	Begg CB, Orlow I, et al	6887	33	Common polymorphism (nt500 and/or nt540 in the 3 ′untranslated region and Ala148Thr in exon 2)
Greek	Nikolaou V, Kang X,et al	320	16	Most common mutation is :p16 p.R24P -34G>C c.41_43delins20bp c.301G>C (p.G101R) c.301G>A (p.G101E) c.296_297insGACC
Australia, Spain and the United Kingdom	MarkHarland, Anne E Cust,et al	2929	58	p.A148T (rs3731249)
Dutch	Gruis NA, van der Velden PA,et al	15	13	19 basepair (bp) germline deletion
Leiden	de Snoo FA, Bishop DT,et al	22 families (1528persons)	209	r.225-243del
Canada	Mark Harland ; Elizabeth A. Holland, et al	107	4	G-34T located in the 5' UTR
United Kingdom	Liu L, Dilworth D,et al	50-80%	25-60%	G-34T (AUG translation initiation codon)
southern Sweden	Åke Borg, Ulla Johansson,et al	10 families	3	in-frame 3-bp insertion (duplication) at nucleotide 332: resulting in an arginine insertion at codon 105
Western Sweden	Erlandson A, Appelqvist F,et al	107 patients (from 68 families)	2	Asp108 Tyr
Canada	W. D. Foulkes, T. Y. Flanders,et al	25families	3	Ins 111Arg

**Table 4:** Residue Conservation

Human	EGFLDTLVVLHRAGARLDVRDAWGRLPVDLAEELGHRDVAR
Mouse	EGFLDTLVVLHGSGARLDVRDAWGRLPLDLAQERGHQDIVR
Rat	EGFLDTLVVLHQAGARLDVRDAWGRLPLDLALERGHHDVVR

## Discussion

Germline CDKN2A mutations ocuur in 5-20% familial melanoma cases (Helgadottir et al., 2014; Helgadottir et al., 2015). This mutation is associated with enhanced risk of melanoma (Helgadottir et al., 2016). Germline mutations in exon 1 CDKN2A affect just the transcript of p16, while a couple of such mutations in exon 2 have the capability to affect both proteins(p16 and p14) (Jenkins et al., 2013). Totally, 40 percent of mutations in CDKN2A occurr in exon 1α and 53% in exon 2. Mainly, mutations in CDKN2A are missense (distributed along the whole length of coding sequences that caused loss of function proteins) or nonsense, insertions or deletions, splicing mutations, and eventually regulatory mutations (70%, 23%, 5%, and 2%, respectively) (Aoude et al., 2015; Potrony et al., 2015). Several mutations are known in this gene. There are many studies related to CDKN2A mutation, some of which are summarized in Table 3. (Gruis et al., 1995; Borg et al., 1996; Foulkes et al., 1997; Aitken et al., 1999; Liu et al., 1999; Harland et al., 2000; Begg et al., 2005; Erlandson et al., 2007; de Snoo et al., 2008; Nikolaou et al., 2011; Harland et al., 2014; Burgstaller-Muehlbacher et al., 2015; Cossu et al., 2016). 

The present study showed that among cases which attended to the Imam Khomeini hospital complex during 10 years, 30 % (92/322) had early onset melanoma. In this study, the patients were checked for mutation in CDKN2A gene. There might be more number of cases if the family history was included; however, communication problems (data collection was done during 10 years) and considering only early onset as a criteria for family melanoma, limited the number of cases. Out of 20 cases, only one of patients had a missense mutation in exon 2 c.322G>C. Although the mutation in this position has been reported before, the acid amino alteration in germ line and in this disease, specifically, is reporting for the first time (as far as the authors are concerned). By using bioinformatics tools (Mutation taster) a pathologic change was observed. Two other reports also pointed the correlation between the mutation in this position and melanoma. One of the studies reported a novel mutation in p16 c.322 G>T; Asp108Tyr (p14Arg163Leu) in three Swedish families (totally 7 cases) which was heterozygous. However, this mutation has been reported as a somatic mutation in other cancers. It was demonstrated that this mutation was involved in pathogenesis of malignant melanoma (Erlandson et al., 2007). Another study had reported the known germline mutation of CDKN2A (322G>A: Asp108Asn) in Australian population and its role in melanoma predisposition (Foulkes et al., 1997). 

In the present study, amongst 20 patients, there was a patient with novel germline missense mutation in CDKN2A (322G>C: Asp108His) (Figure 4). In this substitution, an acidic amino acid (Asp) was replaced by a polar amino acid (His) (Asp) (Erlandson et al., 2007). Aspartate was located on 10th beta turns (Figure 2) in domain 1 of P16(Figure 2) (Foulkes et al., 1997; Fogarty and Bergmann, 2017). This site is highly conservative in human, mouse, and rat (Table 4) (Erlandson et al., 2007). The high conservation may indicate the critical function of p16 protein as a cell cycle regulatory. The domain has caspase 3 and caspase 7 cleavage sites which are well-known enzymes for programmed cell death (Apoptosis) (Panneer Selvam et al., 2018). Apoptosis and proliferation are two opposite points in the cell life. Usually, the balances between these two phenomena are very critical for cell and tissue survival and maintenance (Woo et al., 2003; Fogarty and Bergmann, 2017; Panneer Selvam et al., 2018). It is suggested that a germ line mutation in the caspase cleavage site of CDKN2A could increase the susceptibility to neoplasia. 

The family history of proband revealed a gastric cancer on his mother side (uncle) increasing the possibility of inheritance in this family. Familial melanoma and CDKN2A mutation could be associated with different type of cancers, especially pancreas. Fortunately, there was no mutation in his offspring (two daughters) (Figure 4). Other family members did not attend to screening program, but they were asked to join annually clinical screening program due to increased risk of melanoma in the family. Concerning gastric cancer, there are some reports for the association of this cancer in the family with melanoma and CDKN2A gene mutation (Read et al., 2016).

Familial cancer genes increase the susceptibility of cancer in inherited person among the family member; therefore, it is important to inform family members of the screening advantages in preventing this disease which would save lives and increase the quality of life especially in poor prognostic cancers(Leachman et al., 2017). 

*Study limitation*

This study was conducted according to accepted ethical standard of ethics committee, Tehran University of Medical Sciences. The mutation analysis was limited to the patient and his offspring. Other family members especially parents of patient were refused to participate in sampling and mutation investigation. The parental screening could be helpful to find De novo mutation in proband and increasing the possibility of pathogenic feature in founded mutation 

## Author Contribution Statement

The authors confirm contribution to the paper as follows: study conception and design: Reza Shirkoohi; data collection: Samria Ferdosi and Rezieh Alishahi; analysis and interpretation of results: Reza Shirkoohi for genetic pedigree and DNA sequencing, Alireza Ghanadan for Slide preparation and pathologic diagnosis; draft manuscript preparation: Reza Shirkoohi and Mojtaba saffari. All authors reviewed the results and approved the final version of the manuscript. 
